# Correlation between SEPS1 gene polymorphism and type 2 diabetes mellitus: A preliminary study

**DOI:** 10.1002/jcla.22967

**Published:** 2019-07-02

**Authors:** Feng Li, Aiyou Mao, Xianxian Fu, Yang She, Xiaobin Wei

**Affiliations:** ^1^ Department of Clinical Laboratory Haikou People’s Hospital, Affiliated Haikou Hospital, Xiangya School of Medicine, Central South University Haikou Hainan China; ^2^ Department of Clinical Laboratory Hainan Provincial People's Hospital Haikou Hainan China

**Keywords:** diabetic nephropathy, healthy controls, SEPS1 gene polymorphism, Type 2 diabetes mellitus

## Abstract

**Background:**

The protein encoded by the selenoprotein S gene is considered to be an anti‐inflammatory and antioxidant protein and is involved in a variety of diseases. Therefore, we want to study the distribution characteristics of this gene in Chinese diabetic population.

**Methods:**

A total of 170 patients with DM (including 100 patients with T2DM and 70 patients with diabetic nephropathy [DN]) and 100 healthy controls (HC) were selected from Haikou People's Hospital (China) between January 2017 and July 2017. The polymorphisms of three SEPS1 genes (SNP ID: rs4965814, rs28665122, and rs34713741) were measured by massARRAY method, while the polymorphisms of SEPS1 genes (SNP ID: rs4965373) were detected by Sanger sequencing.

**Results:**

Comparing three groups, the results were the following: (a) There was a significant difference in the genotype and allele distribution of rs34713741 between DN group and HC group and between T2DM group and DN group; For this gene locus, the risk of diabetic nephropathy in healthy individuals with T allele was 0.6 times higher than that in individuals with GG genotype (OR = 0.60, 95% CI: 0.46 ~ 0.77). (b) There was a significant difference in the distribution of rs4975814 genotype between DN group and HC group; for this gene locus, the risk of diabetic nephropathy in healthy individuals with T allele was 2.71 times higher than that in individuals with GG genotype (OR = 2.71, 95% CI: 1.66 ~ 4.45).

**Conclusion:**

We conclude that rs34713741 (GT + TT) may be a protective gene for DN and the rs4975814 (GT + TT) may be a susceptibility gene for DN.

## 1 INTRODUCTION

Type 2 diabetes mellitus (T2DM) is a type of systemic metabolic disorder characterized by absolute or relative reduction in insulin secretion (or insulin resistance) caused by defects in islet β‐cell function, which may occur as a result of genetic, environmental, and immune factors. T2DM is often asymptomatic, and it accounts for approximately 90% of all diagnosed cases of diabetes in adults.

So far, many studies have reported the association between gene polymorphisms and T2DM risk. Selenium is an essential trace element in mammals, which contributes to the normal functioning of multiple systems, including immunity, redox regulation, and resistance to inflammation, through "selenide" glutathione peroxidase and thioredoxin reductase.^1^ Currently, it has been found that selenoprotein gene polymorphism is closely associated with inflammatory and immune diseases. Over the recent years, five kinds of selenoproteins, that is, glutathione peroxidase (GPx), thioredoxin protein reductase 2 (TrxR2), selenoprotein (relative molecular mass 15 000; SEP15), selenoprotein P (SelP), and selenoprotein S (SEPS1, also known as SelS) have been intensively studied. As a transmembrane protein,^2^ SEPS1 constitutes a reverse transport channel for endoplasmic reticulum (ER)‐associated protein degradation together with ER‐associated protein 1, which reversely transports misfolded proteins from the ER to the cytoplasm for degradation, and also inhibits and regulates the inflammatory and immune response caused by misfolded proteins in the ER.^3^ In the human genome, 25 selenoprotein genes have been identified.^4^ In 2002, SEPS1 was discovered as a novel gene that produces a protein that interacts with serum amyloid protein (SAA). SAA is an acute phase protein involved in insulin resistance, which has an important role in the development of diabetes and atherosclerosis.^5^ So far, there have been few reports on the polymorphism of SEPS1 gene in T2DM, especially in the Chinese population.

The following study investigates the correlation between SEPS1 gene polymorphism and T2DM in Chinese population.

## 2 MATERIALS AND METHODS

This study was approved by the Ethics Committee of Haikou People's Hospital (Project Name: The study of inflammatory pathogenesis between selenoprotein S gene polymorphism and type 2 diabetes [ZDYF 2018132], batch number: 2017‐[ethical review] ‐108). All subjects have read and signed the informed consent.

### 2.1 Patients

A total of 100 patients with T2DM, 70 patients with diabetic nephropathy and 100 healthy controls were selected from Haikou People's Hospital (China) between October 2017 and January 2018. The information that could identify individual participants during or after data collection can be access to by us. The specific clinical data for each patient are shown in Table 1 and Table 2. The inclusion criteria for patients with T2DM and diabetic nephropathy were as follows: (a) The T2DM was diagnosed according to the criteria of American Diabetes Association (ADA) in 2016 6 and (b) clinical diagnosis of T2DM or diabetic nephropathy. Healthy controls with primary diseases of important organs (heart, liver, kidney, brain), or with history of drug abuse or allergic constitution were excluded from the study.

**Table 1:** General information of the HC group and DM group

Variables	The HC group (n = 100)	The DM group (n = 170)	*χ* ^2^/t value	*P* value
(Male: female)/n	49:51	84:86	0.004	0.948^a^
Ages/(y)	39.97 ± 5.96	59.8 ± 11.06	−19.133	0.000^b^
The proportion of family history of diabetes	7/100	23/147	2.718	0.099^a^
The proportion of smokers	31/100	49/121	0.450	0.503^a^
BMI body mass index/(kg/m^2^)	23.70 ± 1.34	24.17 ± 2.97	−1.778	0.077^b^

Note

*P* value: a meant chi‐square test and b meant *t* test.

Abbreviations: DM, Diabetes mellitus; HC, Healthy controls.

**Table 2:** General information of the DN group and T2DM group

Variables	The T2DM group (100)	The DN group (70)	*χ* ^2^/t value	*P* value
(Male: female)/n	49:51	35:35	−3.713	1.000^a^
Ages/(y)	57.26 ± 11.36	63.43 ± 9.57	0.000	0.000^b^
The proportion of family history of diabetes	13/100	10/70	0.058	0.809^a^
The proportion of smokers	29/100	20/70	0.105	0.746^a^
BMI body mass index/(kg/m^2^)	24.06 ± 1.71	24.30 ± 1.54	−0.923	0.357^b^
Fasting c‐peptide (ng/mL)	1.65 ± 0.98	1.60 ± 1.31	0.271	0.787^b^
Duration (y)	4.86 ± 3.28	13.02 ± 4.75	−12.423	0.000^b^

Note

*P* value: a meant chi‐square test and b meant *t* test.

Abbreviations: DN, Diabetic nephropathy; T2DM, Type 2 diabetes mellitus.

### 2.2 Genomic DNA extraction from peripheral blood

Genomic DNA was extracted from peripheral blood using DNA extraction kit (Beijing Sunbiotech Company); EDTA anticoagulated venous blood. The obtained DNA solution was stored at −20°C.

### 2.3 Sanger method for SEPS1 gene SNP genotyping

PCR was performed using the following primers: rs4965373, 5′‐GTACAGAACTTTGTAAAAACGAAAC‐3′ (forward) and 5′‐CAGTA ATTCG GCAACTCGACTCC‐3′ (reverse). The primers were synthesized by Prime software (Generay Biotech Company). The total volume of the reaction mixture was as follows: 50 μL Master mix, containing 10 μL of 5× Buffer, 4 μL of dNTP mix (2.5 mM each), 1 μL of upstream primer (10 μM), 1 μL of downstream primer (10 μM), 200 ng of genome, and 0.5 μL of PrimeSTAR HS DNA polymerase and ddH2O. The amplification conditions were as follows: (98°C, 5 minutes) 1 cycle, (98°C, 10 seconds, 55°C 10 seconds, 72°C 20 seconds) 30 cycle, (72°C 8 minutes) 1 cycle, and 4°C permanent storage. The PCR product was then digested and purified: The PCR products of 101 were mixed with 1 unit of shrimp alkaline phosphatase and 2 units of nucleic acid exonuclease I, centrifuged at 2611 *g* for 30 seconds, incubated at 37 for 30 minutes, reacted at 80 for 15 minutes, purified, and stored at 4°C. Sequence: Separation of nucleic acids to be measured based on the principle of complementary pairing of bases. The product was then separated by polyacrylamide gel electrophoresis and sequenced by autoradiography.

### 2.4 MassARRAY method for SEPS1 gene SNP genotyping

PCR was performed using the following primers: rs4965814, 5′‐ACGTTGGATGGCAAGCCAGGTTTAGTCTTC‐3′ (forward) and 5′‐ACGTTGGATGGCTCCTACGCACACGTGGC‐3′ (reverse); rs28665122, 5′‐ACGTTGGATGGCTCCTACGCACACGTGGC‐3′ (forward) and 5′‐ ACGTTGGATGCCAAGAGATAGC ACAGAACG‐3′ (reverse); rs34713741, 5′‐ACGTTGGATGAGGTGGACCATCA GAAGCAG‐3′ (forward) and 5′‐ACGTTGGATGTTTCAGTTCCATTTCCCCCG‐3′ (reverse). All primers were synthesized by Prime software (Generay Biotech Company). The total volume per N reaction mixtures was 4 × n (1 + 20%) μL, containing Water HPLC grade 2.8 × n × (1 + 20%) μL, 0.5 μmol/L PCR primer mix 0.5 × n × (1 + 20%) μL, 10× PCR Buffer 0.4 × n × (1 + 20%) μL, 25 mmol/L MgCl_2_ 0.1 × n × (1 + 20%) μL, 25 mmol/L dNTP mix 1.0 × n × (1 + 20%) μL, 5 U/μL PCR Enzyme 2.8 × n × (1 + 20%) μL, and 10 ng/μL DNA n μL. The amplification conditions were as follows: step 1:95°C 2 minutes; step 2:95°C 30 seconds; step 3:56°C 30 seconds; step 4:72°C 1 minutes; step 5: go to step 2 for 45 cycles; step 6:72°C 5 minutes; and step 7:4°C Permanent storage. Multiple single base extension reactions were following: rs4965814 extension primer, 5′‐CTTGGCTGACCTTAAAC‐3′; rs28665122 extension primer, 5′‐GGACGCGGTCGTGG TCC‐3′; rs34713741 extension primer: 5′‐AGATGGGAAGCTGGGGG‐3′. Extension conditions: step 1:94°C 30 seconds; step 2:94°C 5 seconds; step 3:52°C 5 seconds; step 4:80°C 5 seconds; step 5: go to step 3 for 5 cycles; step 6: go to Step 2 for 40 cycles; step 7:72°C 3 minutes; and step 8:4°C permanent storage. After desalination treatment, the samples of resulting product were put into an array of matrix‐coated SpectroCHIP chips (supplied by Sequenom, Inc) for mass spectrometric detection.

### 2.5 Statistical analysis

SPSS 21.0 statistical software was used for data processing and statistical analysis. The frequency of each allele and genotype of SEPS1 was calculated. Hardy‐Weinberg equilibrium test and linkage disequilibrium test were carried out with SHEsis software to check whether each group accorded with the equilibrium. The SEPS1 gene polymorphism between the groups was compared by independent sample chi‐square test. *P* < 0.05 was considered statistically significant.

## 3 RESULTS

In the study of healthy control group and diabetes mellitus group, Hardy‐Weinberg equilibrium test showed that there was no significant difference in the theoretical frequency and observation frequency of the four Selenoprotein S loci genotypes in diabetes mellitus group, and there was no significant difference in the theoretical frequency and observation frequency of the other three loci except rs28665122 in healthy people, which was consistent with Hardy‐Weinberg equilibrium random mating group. After multivariate logistic regression (MLR) analysis, model fitting significance of the polymorphism of four locus of SEPS1 gene was found all are greater than 0.05 (*P > 0.05*), indicating that the results are not affected by other factors such as age and gender.

Compared to the control group, no significant difference in the genotype and allele distribution was found for rs4965373, rs28665122, rs34713741, and rs4975814 of the DM group (Table 3 and Table 4).

**Table 3:** Genotype and allele distribution of rs4965373 polymorphism in DM group and HC group

Gene locus	The DM group (170)/*P*_H‐W_		The HC group (100)/*P*_H‐W_		*χ* ^2^	*P* value
rs4965373 polymorphisms						
Genotype						
CC	64		48			
CT	90	0.050	45	0.413	2.851	0.240
TT	16		7			
Alleles						
C	218		141		2.303	0.129
T	122		59			

Note

*P* value: chi‐square test.

Abbreviations: DM, Diabetes mellitus; HC, Healthy controls.

**Table 4:** Genotype and allele distribution of three other gene polymorphisms in DM group and HC group

Gene locus	The DM group (123)/*P*_H‐W_		The HC group (75)/*P*_H‐W_		*χ* ^2^	*P* value
rs28665122 polymorphisms						
Genotype						
CC	57		28			
CT	45	0.027	33	0.445	1.598	0.450
TT	21		14			
Alleles						
C	159		89		1.119	0.290
T	87		61			
rs34713741 polymorphisms						
Genotype						
GG	31		21			
GT	67	0.307	43	0.147	0.792	0.673
TT	25		11			
Alleles						
G	130		85		0.548	0.459
T	116		65			
rs4975814 polymorphisms						
Genotype						
GG	20		19			
GT	70	0.094	32	0.216	4.215	0.122
TT	33		24			
Alleles						
G	110		70		0.143	0.705
T	136		80			

Note

In some samples, the amount of DNA remaining was insufficient after first generation sequencing of Sanger, and the cases of actual measurement were 198. *P* value: chi‐square test.

Abbreviations: DM, Diabetes mellitus; HC, Healthy controls.

Next, we compared the difference in the genotype and allele distribution between three groups (T2DM, DN, and HC). Briefly, we found no significant differences in the genotypes and alleles distribution for rs4965373 and rs28665122 between groups. Moreover, statistically significant difference in the genotype and alleles distribution of rs34713741 was found between DN group and HC group (*χ* ^2^ = 6.495, *P* = 0.039 < 0.05; *χ* ^2^ = 5.807, *P* = 0.016 < 0.05); the distribution of genotype and alleles of rs34713741 polymorphism in DN group was significantly different from T2DM group (*χ* ^2^ = 16.370, *P* = 0.000 < 0.05; *χ* ^2^ = 3.630, *P* = 0.000 < 0.05), while there was no difference between the HC group and T2DM group. For this gene locus, the risk of diabetic nephropathy in healthy individuals with T allele was 0.6 times higher than that in individuals with GG genotype (OR = 0.60, 95% CI: 0.46 ~ 0.77). In addition, there was an obvious difference in the distribution of rs4975814 genotype between DN group and HC group (*χ* ^2^ = 8.001, *P* = 0.018 < 0.05), but between T2DM group and HC group, or DN group and T2DM group, while no statistically significant difference was detected in genotype and allelic distribution (Table 5 and Table 6); for this gene locus, the risk of diabetic nephropathy in healthy individuals with T allele was 2.71 times higher than that in individuals with GG genotype (OR = 2.71, 95% CI: 1.66 ~ 4.45). The linkage disequilibrium test showed that there was no linkage disequilibrium between rs34713741 and rs4975814 (*D*′=0.126 < 0.500).

**Table 5:** Comparison of genotypes and alleles distribution of rs4965373 polymorphism between two groups of T2DM and DN and HC groups

Gene locus	The T2DM group (100)	The DN group (70)	The HC group (100)	*χ* ^2^	*P* value
rs4965373					
Genotype					
CC	41	23	48	$\chi_{1}^{2}$ = 1.536	*P*_1_ = 0.464
CT	48	42	45	$\chi_{2}^{2}$ = 4.072	*P*_2_ = 0.131
TT	11	5	7	$\chi_{3}^{2}$ = 2.496	*P*_3_ = 0.287
Alleles					
C	130	88	141	$\chi_{1}^{2}$ = 1.384	*P*_1_ = 0.239
T	70	52	59	$\chi_{2}^{2}$ = 2.188	*P*_2_ = 0.139
				$\chi_{3}^{2}$ = 0.164	*P*_3_ = 0.685

Note

$\chi_{1}^{2}$ and *P*_1_ were compared between T2DM and HC groups; $\chi_{2}^{2}$ and *P*_2_ were compared between DN and HC groups; $\chi_{3}^{2}$ and *P*_3_ were compared between T2DM and DN groups. *P* value: chi‐square test.

Abbreviations: DN, Diabetic nephropathy; HC, Healthy controls; T2DM, Type 2 diabetes mellitus.

**Table 6:** Comparison of genotypes and alleles distribution in three other gene locus polymorphisms between two groups of T2DM and DN and HC groups

Gene locus	The T2DM group (78)	The DN group (45)	The HC group (75)	*χ* ^2^	*P* value
8665122					
Genotype					
CC	33	24	28	$\chi_{1}^{2}$ = 0.743	*P*_1_ = 0.690
CT	29	16	33	$\chi_{2}^{2}$ = 3.167	*P*_2_ = 0.205
TT	16	5	14	$\chi_{3}^{2}$ = 2.247	*P*_3_ = 0.325
Alleles					
C	95	64	89	$\chi_{1}^{2}$ = 0.078	*P*_1_ = 0.780
T	61	26	61	$\chi_{2}^{2}$ = 3.376	*P*_2_ = 0.066
				$\chi_{3}^{2}$ = 2.605	*P*_3_ = 0.107
rs34713741					
Genotype					
GG	11	20	21	$\chi_{1}^{2}$ = 4.536	*P*_1_ = 0.104
GT	52	15	43	$\chi_{2}^{2}$ = 6.495	*P*_2_ = 0.039
TT	15	10	11	$\chi_{3}^{2}$ = 16.370	*P*_3_ = 0.000
Alleles					
G	74	65	85	$\chi_{1}^{2}$ = 2.610	*P*_1_ = 0.106
T	82	25	65	$\chi_{2}^{2}$ = 5.807	*P*_2_ = 0.016
				$\chi_{3}^{2}$ = 14.267	*P*_3_ = 0.000
rs4975814					
Genotype					
GG	14	5	19	$\chi_{1}^{2}$ = 1.588	*P*_1_ = 0.452
GT	40	31	32	$\chi_{2}^{2}$ = 8.001	*P*_2_ = 0.018
TT	24	9	24	$\chi_{3}^{2}$ = 3.630	*P*_3_ = 0.163
Alleles					
G	68	41	70	$\chi_{1}^{2}$ = 0.292	*P*_1_ = 0.589
T	88	49	80	$\chi_{2}^{2}$ = 0.028	*P*_2_ = 0.867
				$\chi_{3}^{2}$ = 0.089	*P*_3_ = 0.765

Note

$\chi_{1}^{2}$ and *P*_1_ were compared between T2DM and HC groups; $\chi_{2}^{2}$ and *P*_2_ were compared between DN and HC groups; $\chi_{3}^{2}$ and *P*_3_ were compared between T2DM and DN groups. In some samples, the amount of DNA remaining was insufficient after first generation sequencing of Sanger, and the cases of actual measurement were 198. *P* value: chi‐square test.

Abbreviations: DN, Diabetic nephropathy; HC, Healthy controls; T2DM, Type 2 diabetes mellitus.

## 4 DISCUSSION

The SEPS1 gene, which is located on 15q26.3, contains 6 exons and 5 introns.^7^ It is expressed in different tissues, including adipose tissue, muscle, and liver.^5^, ^8^ SEPS1 protein is mainly located on the ER membrane surface and cell membrane, and its main biological function is to protect cells from oxidative stress damage, regulate inflammatory response, and degrade endoplasmic reticulum‐associated proteins.^9^ If the SEPS1 gene is mutated, it may affect the normal function of its protein, thereby reducing the body's resistance to inflammatory response. In our study, the polymorphism of four gene loci of SEPS1 was investigated in diabetic (T2DM and DN) and healthy controls. Briefly, significant difference in rs34713741 and rs4965814 polymorphism was found in DN group and T2DM group compared with control group. Based on these results, we concluded that the genotype and allele frequency of rs34713741 locus and genotype frequency of rs4975814 locus in SEPS1 gene were associated with DN. In addition, our data suggested that rs34713741 (GT + TT) may be a protective gene for DN and the rs4975814 (GT + TT) may be a susceptibility gene for DN. In the recruitment of our experimental population, we have to admit that due to lack of consideration, we found that the population between HC and DM group, the average age was significantly different; this may have an impact on the analysis of the results.

Olsson et al^10^ have found that the SEPS1 C3705T polymorphism and the A5227G polymorphism (rs4975814 polymorphism and rs4965373 polymorphism) were associated with serum insulin resistance in Swedish population with chronic metabolic diseases. In our study, no differences in SEPS1 rs4965373 polymorphism were found between diabetic and HC group; however, significant differences in rs4975814 polymorphism were observed between diabetic (mainly in DN group) and healthy populations. Compared with Olsson's research, which was focused on the populations with chronic diseases, possibly caused by multiple factors, this study was dedicated on investigating a single disease population with fewer influencing factors.

The prevalence of genetic polymorphisms may remarkably differ among ethnic groups. Martínez et al^11^ have compared the genotype frequencies of six types of SEPS1 SNPs between type 1 DM (T1DM) patients (n = 311) and non‐T1DM healthy controls (n = 550) in the Spanish population. After gender stratification, he found no difference in SEPS1 SNPs rs11327127, rs28665122, rs4965814, rs12917258, rs4965373, and rs2101171 between those groups, which suggested that these SEPS1 SNPs were not risk factors for T1DM. Furthermore, we found no difference in SEPS1 SNP rs34713714 and rs4965814 between T2DM and HC groups; nevertheless, there was a significant difference between DN and HC groups. The reason for this difference may be that the T2DM‐related complications generally occur late, while the role of the SEPS1 SNP gradually manifests in this long‐term chronic process. Cox et al^12^ have conducted a study for risk factor analysis of subclinical cardiovascular disease and death in 1220 European‐Americans with T2DM and have discovered that patients with coronary calcification plaques are strongly associated with SelS SNP, including rs28665122, rs4965814, rs28628459, and rs7178239 while SEPS1 SNP rs12917258 is associated with coronary calcification plaques in T2DM patients. Santos and colleagues have used a real‐time PCR to study 481 patients with Hashimoto's thyroiditis (HT) in Portugal. They have found a significant correlation between SEPS1‐105GA and AA genotypes and HT, where the proportion of carrying A allele was significantly higher in HT group compared with healthy group. Besides, the rate of male patients carrying A allele was significantly higher compared with the female patients.^13^ On the contrary, Li and his team have performed a study of HT patients in China and have found that 2 SEPS1 SNPs (rs2009895‐rs28665122) were significantly associated with female HT, but not in male population,^14^ confirming the propulsive role of this gene in chronic inflammation. The expression product of the SEPS1 gene also revealed to have an important role in the progression of diabetes. Studies have shown that the expression of SEPS1 in omental adipose tissue of patients with T2DM was higher than that of non‐diabetic patients, and Pearson's correlation analysis confirmed that the expression of SEPS1 in omental adipose tissue was positively correlated with SAA and HOMA‐IR.^15^ These data suggested that expression of SEPS1 in visceral adipose tissue is involved in insulin resistance in patients with T2DM. Moreover, Yu et al^16^ have demonstrated that serum SEPS1 is mainly secreted by hepatocytes, and T2DM patients have lower serum SEPS1 levels compared with healthy controls. His data suggested that the regulation of liver and serum SEPS1 levels might become a new strategy for prevention and treatment of diabetes. Meanwhile, in vitro induction of high‐level SEPS1 expressed by islet β‐cell (Min6) may increase resistance to H_2_O_2_ injury and enhance cell viability, thus suggesting that SEPS1 is a protective factor in islets with the ability to protect islet β cells from oxidative stress damage.^17^ Diabetic nephropathy mainly manifests as impaired glomerular filtration capacity, which is essentially the damage of glomerular capillary endothelial cells. Zhao et al^18^ have transfected SEPS1 overexpression plasmid and SEPS1‐siRNA plasmid into human umbilical vein endothelial cells. After hydrogen peroxide treatment, it was found that overexpression of SEPS1 significantly increases cell viability and superoxide dismutase (SOD) activity, and reduces malondialdehyde (MDA) production and Cav‐1 gene and protein expression. In contrast, decreased expression of SEPS1 significantly reduces cell viability, SOD activity, and PKCα gene and protein expression, and increases MDA production and Cav‐1 gene and protein expression. These data have indicated that SEPS1 protects endothelial cells from oxidative stress by inhibiting the expression of Cav‐1 and PKCα. Hishida et al^19^ have conducted a cross‐sectional data cohort study of polymorphisms of antioxidant enzymes (SOD2, CAT, GPx, TXNRD, SEPP1, SEP15, and SEPS1) in chronic kidney disease patients (CKD) from numerous Japanese institutions, revealing that only CAT C‐262T is correlated with the risk of CKD. Nevertheless, the SEPS1 SNP they studied was mainly 105G‐A (rs34713741), while our study demonstrated that there was a significant association between the polymorphism of rs4713741 and rs4975814 and diabetic nephropathy, which was in line with the observations made by Hishida.

While the cause of diabetes still remains unknown, it is certain that it involves a variety of factors and that it is affected by the external environment and internal genetic factors.

## CONFLICT OF INTEREST

All authors declare no competing interests.

## ETHICAL APPROVAL

All procedures performed in studies involving human participants were in accordance with the ethical standards of the institutional and/or national research committee and with the 1964 Helsinki declaration and its later amendments or comparable ethical standards.
