# Supplementary figures and images for: Correlation between SEPS1 gene polymorphism and type 2 diabetes mellitus: A preliminary study
Source: J Clin Lab Anal. 2019 Jul 2;33(8):e22967. doi: 10.1002/jcla.22967 (PMC6805276; doi:10.1002/jcla.22967)

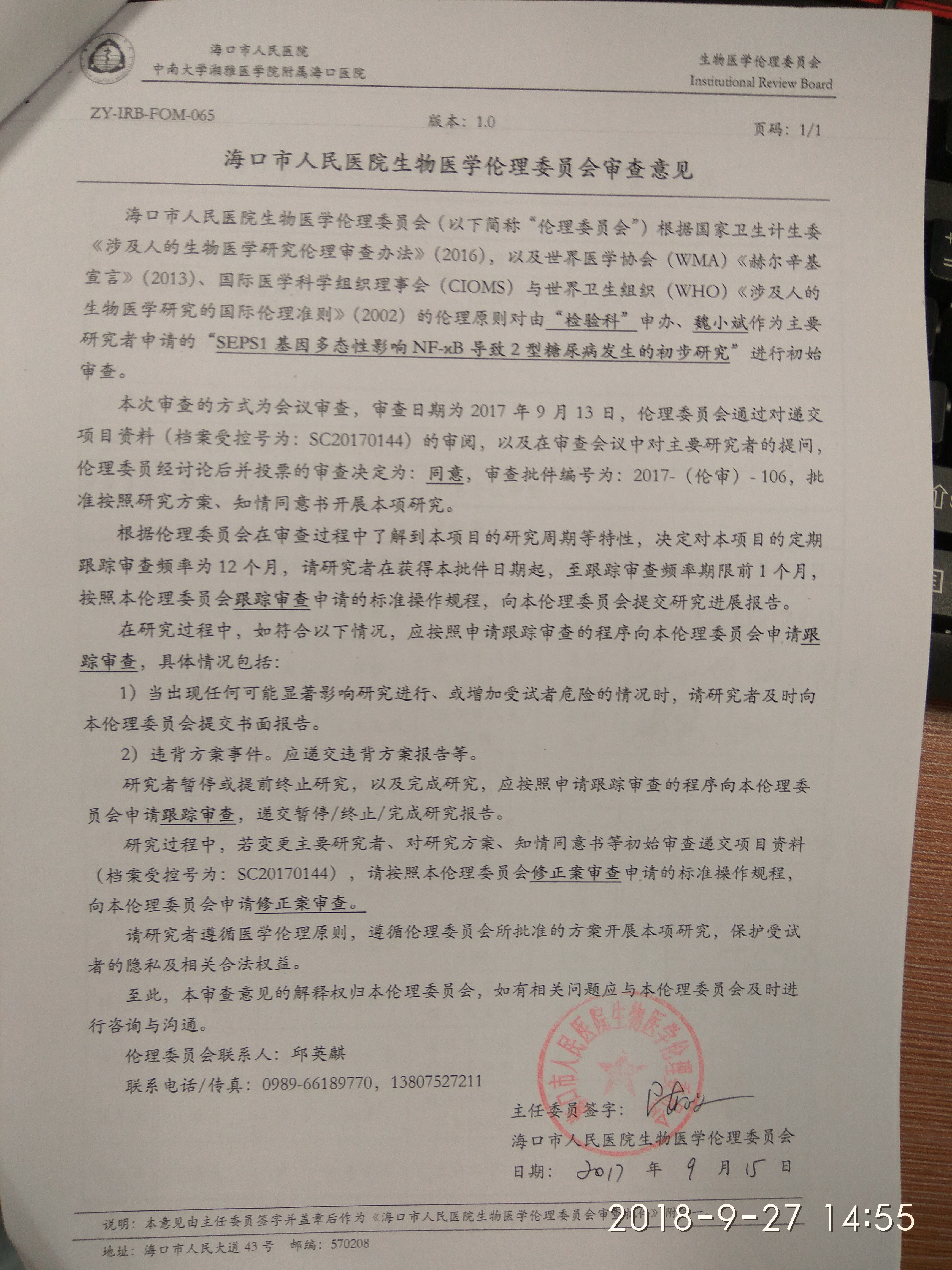

Supplement: Supplementary file 1 [file JCLA-33-e22967-s001.jpg]

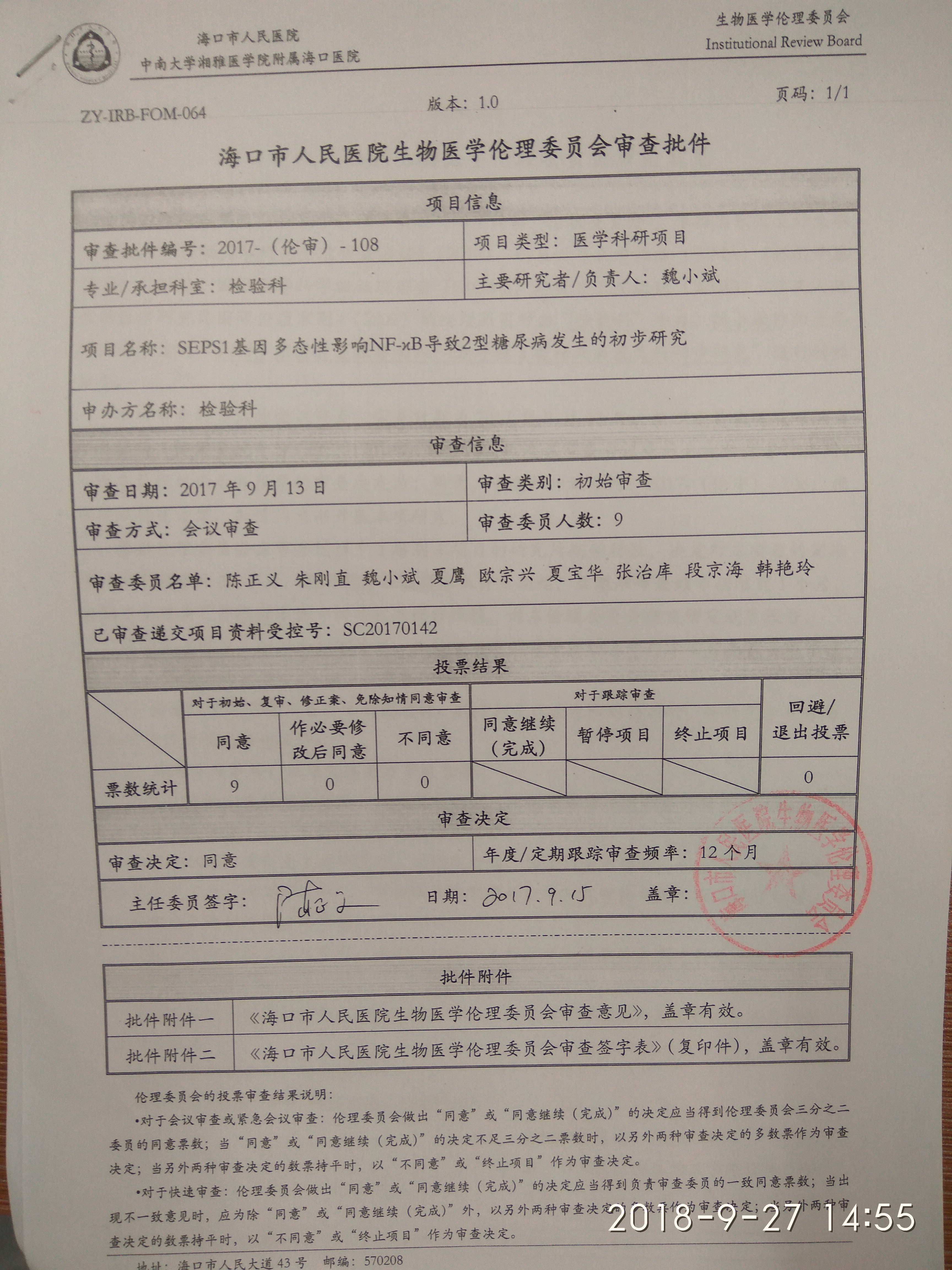

Supplement: Supplementary file 2 [file JCLA-33-e22967-s002.jpg]

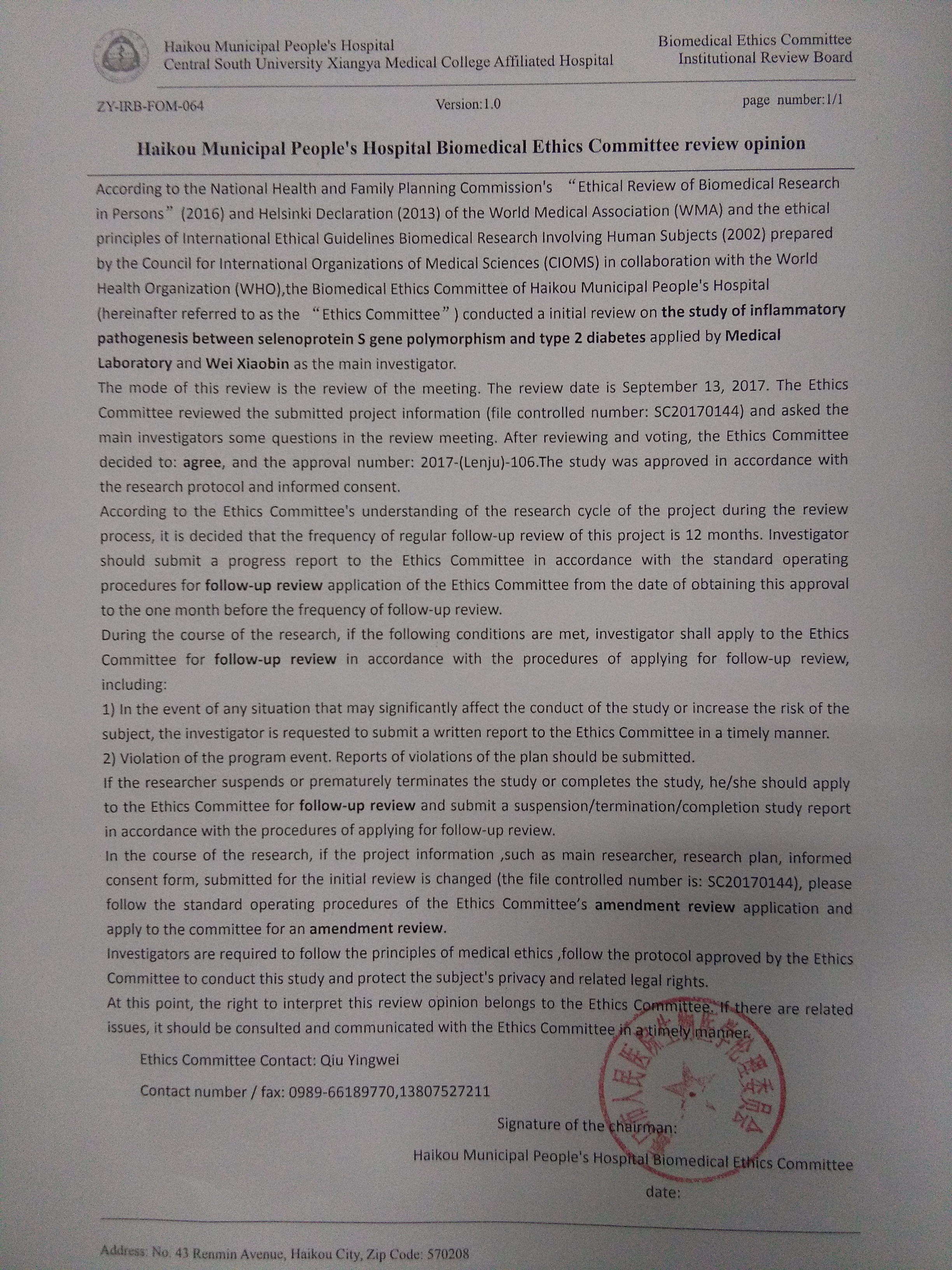

Supplement: Supplementary file 3 [file JCLA-33-e22967-s003.jpg]

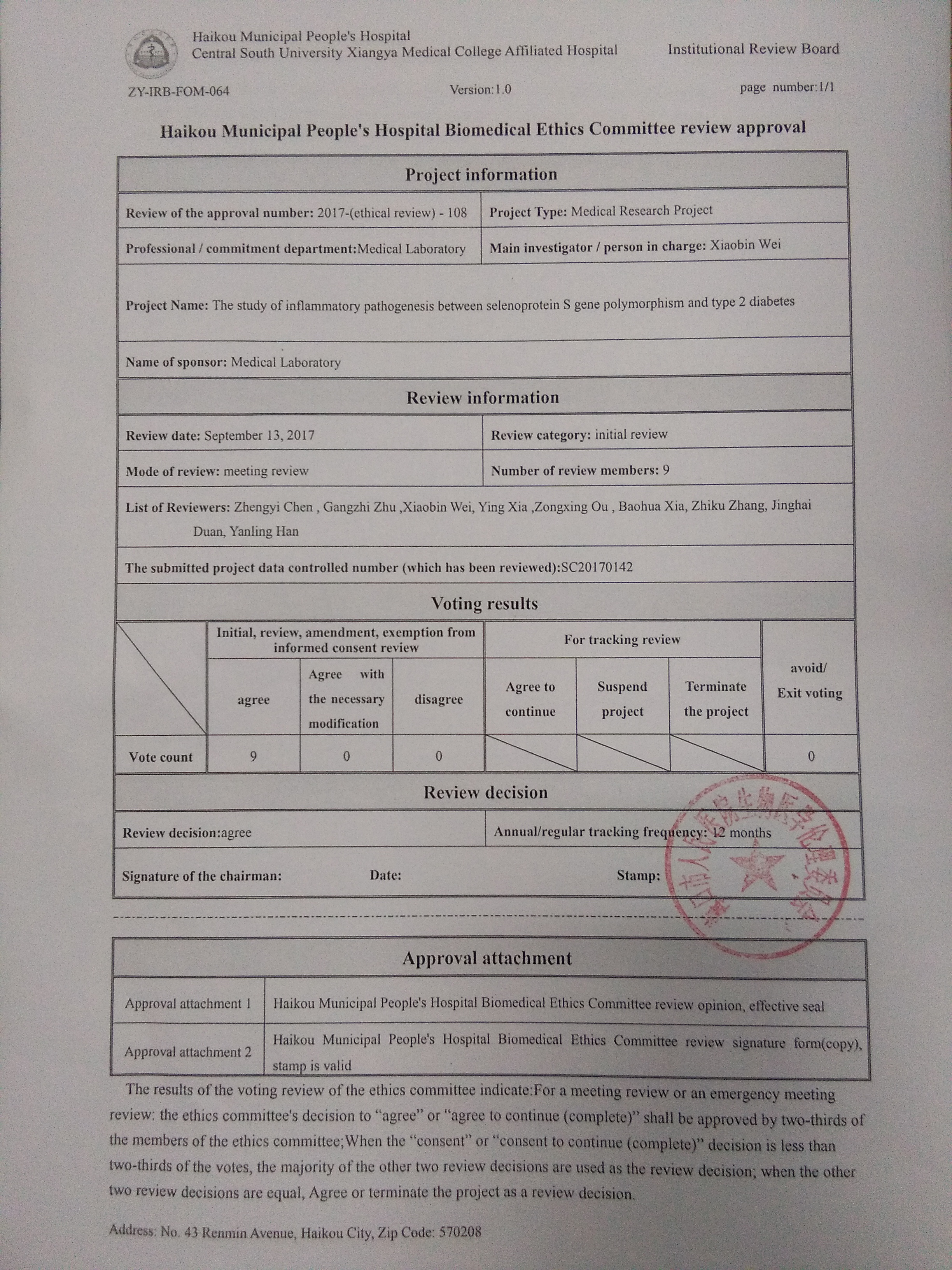

Supplement: Supplementary file 4 [file JCLA-33-e22967-s004.jpg]
